# Clinically significant changes in pain along the Pain Intensity Numerical Rating Scale in patients with chronic low back pain

**DOI:** 10.1371/journal.pone.0229228

**Published:** 2020-03-03

**Authors:** Hidenori Suzuki, Shuichi Aono, Shinsuke Inoue, Yasuaki Imajo, Norihiro Nishida, Masahiro Funaba, Hidenori Harada, Aki Mori, Mishiya Matsumoto, Fumihiro Higuchi, Shin Nakagawa, Shu Tahara, Satoshi Ikeda, Hironori Izumi, Toshihiko Taguchi, Takahiro Ushida, Takashi Sakai

**Affiliations:** 1 Department of Orthopaedics Surgery, Yamaguchi University Graduate School of Medicine, Yamaguchi, Japan; 2 Pain Management Research Institute, Yamaguchi University Hospital, Yamaguchi, Japan; 3 Multidisciplinary Pain Center, Aichi Medical University, Nagakute, Aichi, Japan; 4 Department of Pain Data Management, Aichi Medical University, Nagakute, Japan; 5 Department of Anesthesiology, Yamaguchi University Graduate School of Medicine, Yamaguchi, Japan; 6 Department of Psychiatry, Yamaguchi University Graduate School of Medicine, Yamaguchi, Japan; 7 Department of Rehabilitation, Yamaguchi University Hospital, Yamaguchi, Japan; 8 Department of Orthopedics Surgery, Yamaguchi Rosai Hospital, Yamaguchi, Japan; Cleveland Clinic, UNITED STATES

## Abstract

Low back pain (LBP) is the most common cause of chronic pain. Numerous clinical scales are available for evaluating pain, but their objective criteria in the management of LBP patients remain unclear. This study aimed to determine an objective cutoff value for a change in the Pain Intensity Numerical Rating Scale (ΔPI-NRS) three months after LBP treatment. Its utility was compared with changes in six commonly used clinical scales in LBP patients: Pain Disability Assessment Scale (PDAS), Pain Self-Efficacy Questionnaire (PSEC), Pain Catastrophizing Scale (PCS), Athens Insomnia Scale (AIS), EuroQoL 5 Dimension (EQ5D), and Locomo 25. We included 161 LBP patients treated in two representative pain management centers. Patients were partitioned into two groups based on patient’s global impression of change (PGIC) three months after treatment: satisfied (PGIC = 1, 2) and unsatisfied (3–7). Multivariate logistic regression analysis was performed to explore relevant scales in distinguishing the two groups. We found ΔPI-NRS to be most closely associated with PGIC status regardless of pre-treatment pain intensity, followed by ΔEQ5D, ΔPDAS, ΔPSEC, and ΔPCS. The ΔPI-NRS cutoff value for distinguishing the PGIC status was determined by ROC analysis to be 1.3–1.8 depending on pre-treatment PI-NRS, which was rounded up to ΔPI-NRS = 2 for general use. Spearman’s correlation coefficient revealed close relationships between ΔPI-NRS and the six other clinical scales. Therefore, we determined cutoff values of these scales in distinguishing the status of ΔPI-NRS≥2 vs. ΔPI-NRS<2 to be as follows: ΔPDAS, 6.71; ΔPSEC, 6.48; ΔPCS, 6.48; ΔAIS, 1.91; ΔEQ5D, 0.08; and ΔLocomo 25, 9.31. These can be used as definitive indicator of therapeutic outcome in the management of chronic LBP patients.

## Introduction

Chronic low back pain (CLBP) has a major impact on the patient’s quality of life. It causes sleep interruption, fatigue, depressed mood, activity limitations, and restrictions in participation [1, 2]. However, pain is a unique experience that no other person can feel or perceive on one’s behalf. In fact, even if a group of individuals receives the same stimuli or undergoes the same intervention, the rating of pain reported by the patients differs greatly.

In the management of CLBP patients, pain intensity is most frequently measured on the 11-point Pain Intensity Numerical Rating Scale (PI-NRS), which ranges from no pain = 0 to the worst possible pain = 10 [3–5]. As additional tools in evaluating the status of chronic pain, many studies rely on patients’ self-administered answers to the questionnaires of various clinical scales, such as health-related quality of life (QOL) questionnaires [6–7], the Pain Disability Assessment Scale (PDAS), Hospital Anxiety and Depression Scale (HADS), Pain Catastrophizing Scale (PCS) [8], and the Athens Insomnia Scale (AIS), a sleep disorder scale [9–15]. Among these clinical scales, the PI-NRS is considered to be the most subjective one in that it depends on the sensitivity to pain of each patient [16]. Therefore, although the PI-NRS itself is not so reliable for the objective assessment of pain intensity, post-treatment change in the PI-NRS (ΔPI-NRS) is generally considered useful in judging the effectiveness of a therapeutic regimen [16]. However, an appropriate threshold (cutoff) value for ΔPI-NRS is not available for clinical use in the field of CLBP management. It has to be based on the patient’s global impression of change (PGIC) in pain intensity [17–19]. Besides, the utility of the cutoff value should be evaluated based on whether it exceeds a minimally clinically important difference (MCID), which can be defined based on the inherent variability of ΔPI-NRS. However, clinical assessment of the utility of ΔPI-NRS specifically targeting CLBP patients has not been undertaken yet [18–20]. In addition, previous papers have not revealed relationships between ΔPI-NRS and changes in the other above-mentioned clinical scales in the management of CLBP.

This study has four purposes: (1) to clarify the utility of ΔPI-NRS in reference to MCID based on the inherent variability of ΔPI-NRS among patients with CLBP, (2) to predict a cutoff value for ΔPI-NRS in distinguishing the status of PGIC: satisfied vs. non-satisfied, (3) to evaluate correlations between ΔPI-NRS and post-treatment changes in other clinical scales, and (4) to determine the threshold values of the other scales in distinguishing the status of ΔPI-NRS: improved vs. non-improved. We systematically investigated these aspects of ΔPI-NRS to verify its utility as a primary measure of pain relief by conducting multifaceted evaluations of CLBP patients who visited two representative pain management centers in Japan.

## Materials and methods

**Fig 1:**
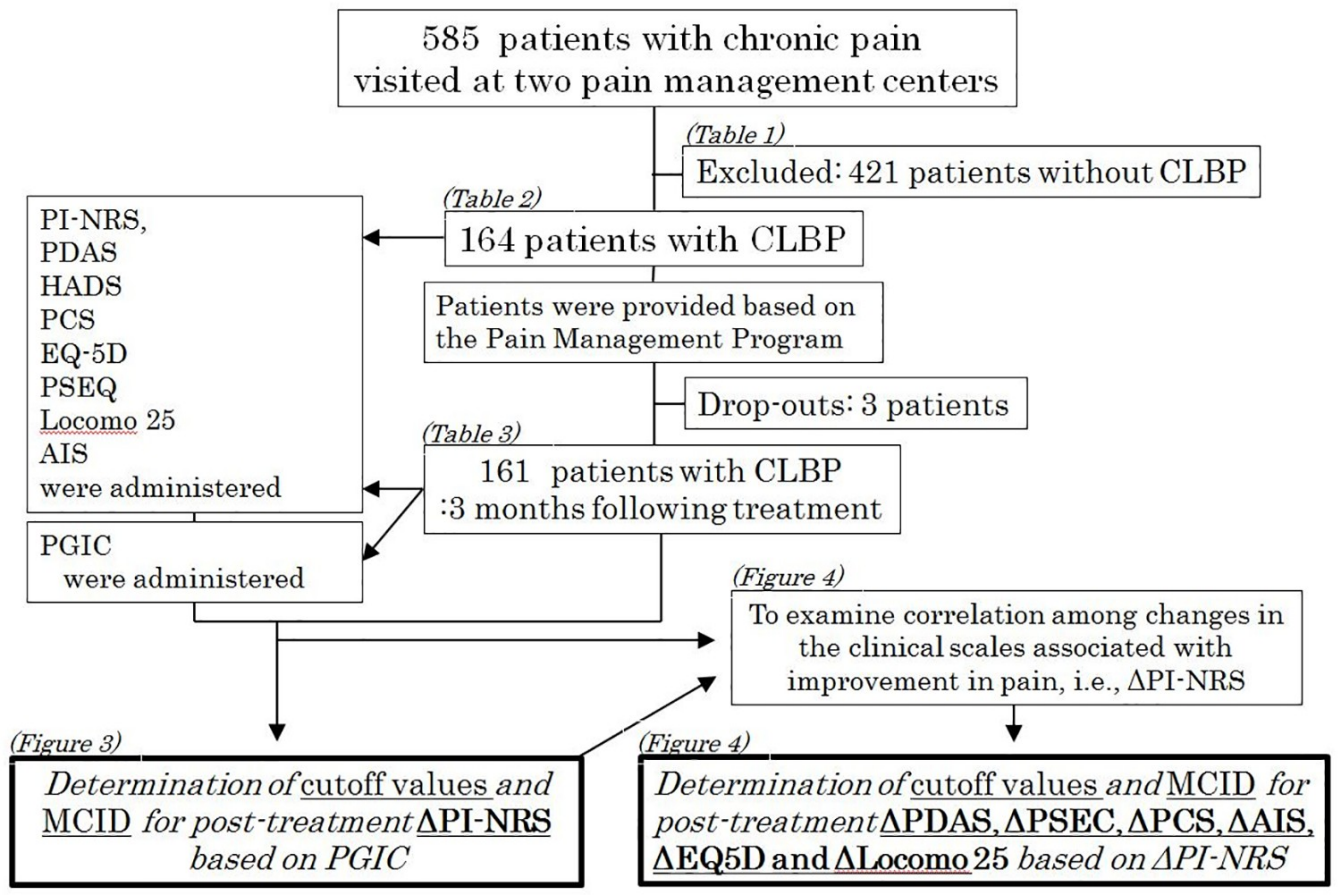
Study flowchart of the study to show how to lead the MCID and the cutoff values. CLBP; Chronic low back pain, PI-NRS; Pain Intensity Numerical Rating Scale, PDAS; Pain Disability Assessment Scale, HADS; The Hospital Anxiety and Depression Scale, PCS; Pain Catastrophizing Scale, EQ5D; EuroQoL 5 Dimension, PSEC; Pain Self-Efficacy Questionnaire, AIS; Athens Insomnia Scale.

This was a cross-sectional study conducted within the usual clinical care of CLBP patients. From among 585 chronic pain patients treated at two pain management centers in Aichi Medical University and Yamaguchi University between 2010 and 2018, 161 patients with CLBP were enrolled in this study after excluding 3 patients who dropped out or failed to complete the questionnaires at the 3-month follow-up. The patients suffered from CLBP with or without radiculopathy due to lumbar spinal stenosis, lumbar disc herniation, myofascial issues, and other causes of pain (Fig 1).

The inclusion criteria in this study was the patients from 20 to 85 years with LBP which was defined as experiencing pain, discomfort and stiffness in the lower back from the 12th rib to the lumbar or lumbosacral area, including lower limb symptoms. Inclusion and exclusion criteria are listed in Table 1. Patients’ demographic data at baseline are presented in Table 2. This study was approved by the Institutional Review Boards of Yamaguchi University (H28-1351) and in Aichi Medical School (No.12-067). All participants provided written informed consent.

**Table 1:** Inclusion and exclusion criteria for enrollment of patients with chronic LBP.

Inclusion criteria	Exclusion criteria
Age 20–85 years	Inability to understand and read the Japanese language, drug abuse, dementia, or other reasons to suspect poor adherence to follow-up
Symptoms of LBP with or without radiculopathy	
Duration of LBP of at least 3 months	

LBP, low back pain.

**Table 2:** Characteristics and baseline clinical profile of chronic low back pain patients with mild, moderate and severe pain before treatment.

Characteristic	All patients (n = 161)	1≤PI-NRS<4: Patients with mild pain (n = 29)	4≤PI-NRS≤6: Patients with moderate pain (n = 81)	7≤PI-NRS≤10: Patients with severe pain (n = 51)
Age (yrs) (mean SD)	59 ± 16.0 (range 20–85)	57 ± 14.0 (range 32–79)	59 ± 16.3 (range 20–85)	59 ± 16.0 (range 26–85)
Gender (Male/Female)	71/90	15/14	33/48	23/28
Duration of pain (months) (mean SD)	75±16.0 (range 3–660)	111±126 (range 3–480)	59±111 (range 3–660)	63±73.2 (range 3–294)
Previous lumbar spine surgery	25	3	15	8
Baseline local PI-NRS (mean SD)	5.54 ± 1.96	2.62 ± 0.71	5.17 ± 0.766	7.78 ± 0.93
PDAS	27.5 ± 10.9	19.7 ± 8.34	26.4 ± 9.24	33.1 ± 11.4
HADS (Anxiety)	8.28 ± 3.86	7.10 ± 3.58	8.28 ± 3.86	9.31 ± 4.05
HADS (Depression)	8.22 ± 4.14	7 ± 4.80	8.22 ± 4.14	9.50 ± 4.48
PCS	35.4 ± 9.53	28.6 ± 8.19	35.4 ± 9.53	39.2 ± 7.91
PCS (Rumination)	13 ± 2.78	11.4 ± 2.69	12.9 ± 2.91	14.0 ± 2.09
PCS (Magnification)	7.19 ± 3.06	5.58 ± 3.01	7.43 ± 3.04	7.72 ± 2.83
PCS (Helplessness)	15.2 ± 5.06	11.5 ± 4.45	15.2 ± 5.03	17.4 ± 4.10
EQ-5D	0.548 ± 0.145	0.657 ± 0.116	0.571 ± 0.104	0.448 ± 0.154
PSEQ	25.4 ± 13.3	31.4 ± 12.4	27.8 ± 12.7	17.9 ± 11.4
Locomo 25	40.5 ± 20.3	24.3 ± 12.9	39.1 ± 16.7	52.0 ± 21.8
Athens insomnia scale	8.74 ± 4.93	6.24 ± 3.71	7.82 ± 4.29	11.6 ± 5.16

PI-NRS, Pain Intensity Numerical Rating Scale; PDAS, Pain Disability Assessment Scale; HADS, Hospital Anxiety and Depression Scale; PCS, Pain Catastrophizing Scale; EQ-5D, EuroQoL 5 Dimension; PSEQ, Pain Self-Efficacy Questionnaire.

### 1.1. Patient care

Therapeutic regimens for CLBP patients were provided based on the Pain Management Program (PMP). PMPs are rehabilitation-based multidisciplinary programs for people with chronic pain [22, 23]. They involve a group of clinicians, nurses, physiotherapists, and psychologists led by pain medicine specialists who collaborate in assisting patients to bring their pain under control. The patients attended PMPs after several therapies had met with limited success. The team of experienced health care professionals at each hospital worked closely with the patients, and, in general, the PMPs were tailored to the individual patient’s clinical needs. The team collaborated in the rehabilitation by providing exercise plans, pharmacotherapy, psychotherapy, cognitive behavioral therapy, patient education, nerve blocks guided by ultrasound or X-ray, and radiofrequency nerve ablation to promote the relief of pain in the rehabilitation program [21].

### 1.2. Scales of multifaceted clinical assessment

For multifaceted clinical assessment of the patients’ conditions, we used the following seven well-established scales:

*Pain Disability Assessment Scale (PDAS)*: A scale for measuring lifestyle disabilities of chronic pain patients. Higher scores (on a scale of 0 to 60 points) indicate greater degrees of lifestyle disability [15].

*The Hospital Anxiety and Depression Scale (HADS)*: A self-reported instrument used to evaluate depression and anxiety in clinical research. The HADS has advantages over other such assessments in that it is efficient in assessing both anxiety and depression. It is composed of 14 questionnaire items and was originally developed for a general medical rather than psychiatric field. Higher scores (0 to 21 points both for anxiety and depression) indicate greater degrees of anxiety and depression [21].

*Pain Catastrophizing Scale (PCS)*: Pain catastrophizing affects how individuals experience pain. The PCS assesses catastrophizing (rumination, magnification, and helplessness) about pain, with higher scores (0 to 52 points) indicating greater degrees of catastrophizing [14].

*Pain Self-Efficacy Questionnaire (PSEQ)*: PSEQ is a 10-item questionnaire developed to assess the confidence of patients with ongoing pain in performing daily activities while in pain. The PSEQ is applicable to any type of persisting pain. It covers a range of functions, including household chores, socializing, work, and coping with pain without medication. Scores can range from 0 to 60, and higher scores indicate greater degrees of performing activities while in pain [11].

*EuroQoL 5 Dimension (EQ-5D)*: The EQ-5D assesses (on a scale of 0 to 1.0) the outcome of health-related aspects of QOL (mobility, self-care, usual activities, pain/discomfort, and anxiety/depression). Zero indicates death and 1.0 indicates complete health [6–7].

*Athens Insomnia Scale (AIS)*: This scale assesses the severity of insomnia using diagnostic criteria set forth by the International Classification of Diseases (ICD-10). The eight-item questionnaire evaluates sleep onset, night and early-morning waking, sleep time, sleep quality, frequency and duration of complaints, distress caused by the experience of insomnia, and interference with daily functioning [13].

*Locomo 25*: This was developed as a screening tool for locomotive syndrome by a Japanese orthopedic surgeon group in 2008. It consists of 25 questions aimed at musculoskeletal disorders such as walking disability, difficulty in daily living, or suffering pain within the body. Scores can range from 0 to 100, and higher scores indicate a greater degrees of performing activities while in pain [10].

### 1.3. Data collection

The PI-NRS, PDAS, HADS, PCS, EQ-5D, PSEQ, Locomo 25, and AIS were administered to 161 patients with CLBP before and 3 months after treatment during 2010 through 2018.

### 1.4. Measurements

Before and 3 months after the therapy, a PI-NRS score reported on a scale of 0−10 was obtained as an indicator of the average level of pain over the past 7 days [22]. For multi-faceted assessments of the chronic LBP patients, the PDAS, HADS, PCS, PSEQ, and AIS were administered twice, once before and once after the therapy, together with PI-NRS. We also administered the Locomo 25 and EQ-5D to assess physical functions (Fig 1).

### 1.5 Patient’s global impression of change

To detect clinically relevant changes in the PGIC, the concept of the transition method was used [17–19]. The transition questionnaire investigates current pain intensity compared to that before treatment. The PGIC was administered at the time of the 3-month follow-up, and patients were asked to score the change on the following scale: (1) much improved, (2) improved, (3) slightly improved, (4) no change, (5) slightly worse, (6) worse, or (7) much worse.[5,14,28]

### 1.6. Statistical analyses for assessment of clinical utility of ΔPI-NRS and changes in other clinical scales

#### 1.6.1. MCID and cutoff value for ΔPI-NRS

The post-treatment change in PI-NRS, or ΔPI-NRS, was calculated as (PI-NRS follow-up − PI-NRS baseline). We sought to predict the MCID and cutoff value for ΔPI-NRS using the anchor-based approach by setting PGIC as the anchor [23]. Therefore, we partitioned LBP patients into two groups based on post-treatment PGIC score: Group 1: satisfied (PGIC = 1 or 2) vs. Group 2: not satisfied (PGIC = 3–7) as was done in previous studies [19, 24–25]. The utility of ΔPI-NRS in distinguishing the two groups were evaluated from satisfied (PGIC = 1 or 2) vs. not satisfied (PGIC = 3–7).

The effect ratio of observed between-group differences was defined as the difference in averages of scale changes (ΔS) or ΔPI-NRS for Group 1 and Group 2, ${\overset{-}{\Delta S}}_{G1},\;{\overset{-}{\Delta S}}_{G2}$, divided by the average within-group variations of ΔS, or SD (ΔS), as shown in the formula:

$$Effect\;ratio=\frac{{\overset{-}{\Delta S}}_{G1}-{\overset{-}{\Delta S}}_{G2}\;}{SD(\Delta S)}.$$

Based on Cohen’s criteria [26], the MCID for ΔPI-NRS was defined as half of the average inherent variability of ΔPI-NRS or 0.5×SD (ΔPI-NRS) [26–28].

Therefore, if ${\bar{\Delta \text{PI}\cdot \text{NRS}}}_{G1}-{\bar{\Delta \text{PI}\cdot \text{NRS}}}_{G2}>0.5\times SD\left(\Delta \text{PI}\cdot \text{NRS}\right)$, we regarded ΔPI-NRS as having clinical utility and proceeded to determine its cutoff value at which the sensitivity of detecting satisfied cases is equal to the specificity of detecting non-satisfied cases. The magnitude of the clinical utility of ΔPI-NRS was expressed as an area under the curve (AUC) of a receiver operating characteristic (ROC) curve.

These analyses were performed for three conditions according to the pre-treatment intensity of pain judged by the PI-NRS: moderate pain (PI-NRS = 4–6), severe pain (7–10), and moderate + severe pain (4–10). In performing the analyses, we excluded 29 LBP patients with mild intensity of pain (PI-NRS = 1–3) because of their lack of sufficient relevance in the evaluation of pain-based treatment effect.

#### 1.6.2 Relationship between ΔPI-NRS and changes in PDAS, HADS, PCS, PSEQ, AIS, Locomo 25, and EQ-5D

The reliability of ΔPI-NRS was evaluated in comparison with post-treatment changes in the other clinical scales of ΔPDAS, ΔHADS, ΔPCS, ΔPSEQ, ΔAIS, ΔLocomo 25, and ΔEQ5D by use of Spearman correlation coefficients. The relative importance of ΔPI-NRS as a marker of pain relief was also assessed by use of multivariate logistic regression analysis by setting the satisfied status of PGIC (scale value of 1 or 2) as a binary objective variable and setting all other clinical scale changes as explanatory variables. We also investigated the clinical implication of the ΔPI-NRS cutoff value in relation to changes in the other clinical scales. For this objective, we partitioned CLBP patients at the cutoff value into two groups and examined how well other clinical scales could distinguish the ΔPI-NRS status by determining respective cutoff values and AUCs based on the ROC analysis.

## Results

Patient characteristics and profiles of 12 clinical scales before and three months after treatment are respectively presented in Tables 2 and 3, separated by mild, moderate, and severe pain groups. The post-treatment levels of patient’s satisfaction by PGIC score are shown in Table 4 in relation to ΔPI-NRS.

**Table 3:** Clinical profile of patients with chronic low back pain 3 months after treatment.

3 months after treatment	*All patients (n = 161)*	1≤PI-NRS<4: *Patients with mild pain (n = 29)*	4≤PI-NRS≤6: *Patients with moderate pain (n = 81)*	7≤PI-NRS≤10: *Patients with severe pain (n = 51)*
PI-NRS (mean SD)	4.15 ± 2.24	2.65 ± 1.84	3.65 ± 1.79	5.76 ± 2.19
PDAS	20.1 士 11.6	15.7 士 10.4	18.2 ± 10.2	25.8 士 11.8
HADS (Anxiety)	8.28 士 3.86	5.79 ± 3.68	6 ± 3.42	8.31 士 4.78
HADS (Depression)	6.68 ± 4.22	6.41 ± 4.67	8.22 ± 4.14	8.35 ± 4.45
PCS	27.3 ± 12.3	23.1 ± 12.3	26.3 ± 12.2	31.3 士 11.0
PCS (Rumination)	10.5 士 4.06	9.37 ± 4.22	10.4 ± 4.27	11.6 ± 3.19
PCS (Magnification)	5.52 士 3.09	4.68 士 3.29	5.25 ± 2.96	6.49 ± 2.93
PCS (Helplessness)	11.1 ± 6.24	9.10 ± 6.26	10.6 ± 5.97	13.2 ± 5.95
EQ-5D	0.647 ± 0.165	0.734 ± 0.151	0.664 士 0.142	0.564 ± 0.165
PSEQ	33.6 ± 13.2	38.8 士 11.1	34.4 ± 12.6	29.3 士 13.7
Locomo 25	29.4 ± 19.5	19.7 ± 17.4	26.0 ± 16.6	40.6 士 19.7
Athens Insomnia Scale	6.30 士 4.16	5.10 ± 3.62	5.51 ± 3.46	8.23 ± 4.75
Patient's satisfaction (3-month follow-up)	**(n = 159)**	**(n = 28)**	**(n = 80)**	**(n = 51)**
Much improved (1)	11	1	8	2
Improved (2)	40	9	22	9
Slightly improved (3)	52	10	28	14
No change (4)	43	5	18	20
Slightly worse (5)	9	3	4	2
Worse (6)	4	0	0	4
Much worse (7)	0	0	0	0

PI-NRS, Pain Intensity Numerical Rating Scale; PDAS, Pain Disability Assessment Scale; HADS, Hospital Anxiety and Depression Scale; PCS, Pain Catastrophizing Scale; EQ-5D, EuroQoL 5 Dimension; PSEQ, Pain Self-Efficacy Questionnaire.

**Table 4:** Relationship between ΔPI-NRS and patient’s satisfaction 3 months after treatment.

	All patients (n = 159)		1≤PI-NRS≤4: Patients with mild pain (n = 28)		4≤PI-NRS≤6: Patients with moderate pain (n = 80)		7≤PI-NRS≤10: Patients with severe pain (n = 51)	
Pre minus post-treatment PI-NRS	Average PI-NRS change (mean SD)	Totals 159	Average PI-NRS change (mean SD)	Totals 28	Average PI-NRS change (mean SD)	Totals 80	Average PI-NRS change (mean SD)	Totals 51
**Patient's satisfaction (3-month follow-up)**								
Much improved (1)	4.36 3.20	11	2	1	4 ± 1.31	8	5 ± 1.41	2
Improved (2)	2.71 ±2.74	40	1.11 ±1.36	9	2.23 ± 1.31	22	3.56 ±2.92	9
Slightly improved (3)	0.94 ±2.62	52	0.1 ± 1.37	10	1±2.07	28	1.79 ±1.63	14
No change (4)	0.02 ±2.99	43	-2.4 ± 2.30	5	0.61 ±1.54	18	1.4 ± 1.73	20
Slightly worse (5)	1 ±2.5	9	-0.67 ± 0.58	3	0.5 ±0.58	4	2 ± 2.83	2
Worse (6)	1 ± 1.41	4		0		0	1 ± 1.41	4
Much worse (7)	0	0		0		0		0

PI-NRS, Pain Intensity Numerical Rating Scale.

### 2.1. Determination of cutoff values and MCID for post-treatment ΔPI-NRS

LBP patients were partitioned into two groups in reference to post-treatment PGIC: satisfied (PGIC = 1–2) and non-satisfied (PGIC = 3–7) groups. The utility of the clinical scales in distinguishing the status of satisfaction by PGIC was explored by the use of multivariate logistic regression analysis in three ways according to the pre-treatment level of pain by PI-NRS: groups of patients with moderate, severe, and moderate + severe pain (Table 5). The final list of clinical scales that were found significant in predicting PGIC satisfaction status as determined by the stepwise selection method are listed in each table.

**Table 5:** Multivariate analyses for the utility of clinical parameters in predicting the status of satisfaction by patient’s global impression of change.

Exp. variables	*β*	SE(*β*)	z	P
M oderate pain (n = 80)				
Sex	-0.327	0.751	-0.436	0.66297
Age	0.031	0.023	1.332	0.18284
ΔPI-NRS	0.963	0.282	3.413	**0.00064**
ΔEQ5D_Q2	2.719	0.957	2.841	**0.00449**
ΔEQ5D_Q5	-1.551	0.837	-1.853	0.06389
ΔHADS_anxiety	-0.506	0.177	-2.851	**0.00436**
ΔHADS_depr	-0.203	0.125	-1.620	0.10521
ΔAIS	0.283	0.132	2.140	**0.03237**
Overall AUC = 0.927				
Severe pain (n = 47)				
Sex	-2.264	1.353	-1.673	0.09436
Age	0.021	0.034	0.611	0.54101
ΔPI-NRS	0.595	0.256	2.330	**0.01981**
ΔEQ5D_Q1	5.602	2.199	2.548	**0.01085**
ΔLocom o 25	0.145	0.066	2.209	**0.02716**
Overall AUC = 0.929				
Moderate + severe pain (n = 127)				
Sex	-0.640	0.526	-1.217	0.22344
Age	0.023	0.016	1.446	0.14819
ΔPI-NRS	0.636	0.163	3.894	**0.00010**
ΔEQ5D_Q2	1.638	0.552	2.970	**0.00298**
ΔEQ5D_Q3	-0.835	0.427	-1.954	0.05076
ΔHADS_anxiety	-0.176	0.083	-2.127	**0.03341**
ΔHADS_depr	-0.246	0.084	-2.915	**0.00356**
ΔAIS	0.154	0.077	1.996	**0.04592**
Overall AUC = 0.883				

For the analysis targeting the moderate and moderate + severe pain groups, ΔPI-NRS was the leading predictor of satisfaction status with P values of 0.00064 and 0.0001, respectively. Other significant predictors among the clinical scales were ΔEQ5D-Q2, ΔHADS-anxiety, and ΔAIS in the analysis of the moderate pain group and ΔEQ5D-Q2, ΔHADS-anxiety, ΔHADS-depr, and ΔAIS in the analysis of the moderate + severe pain group.

In the analysis targeting the severe pretreatment pain group, ΔPI-NRS, ΔEQ5D-Q1, and ΔLocomo 25 showed nearly equal contribution in predicting a satisfactory status.

**Fig 2:**
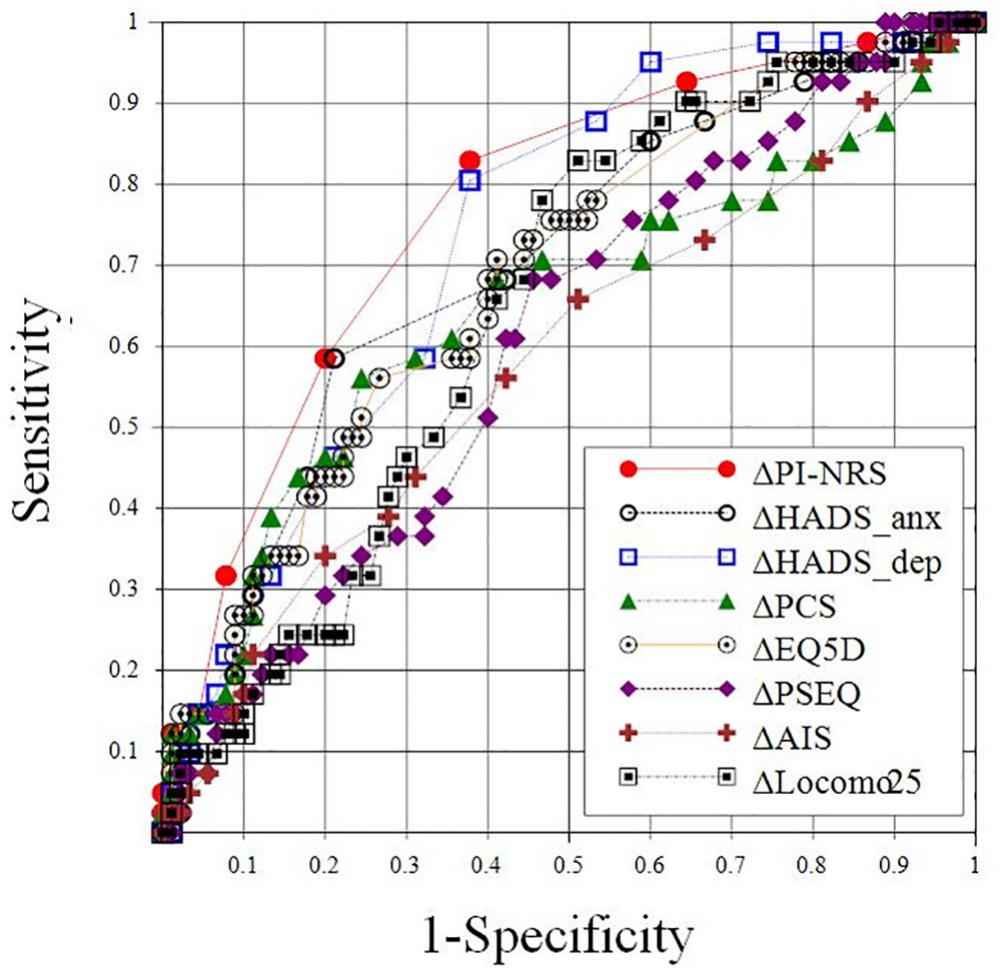
Receiver operating characteristic (ROC) analysis of 8 major clinical scales for their utility in distinguishing the status of satisfaction based on the patient’s global impression of change (PGIC). Post-treatment changes in the 8 major clinical scales were compared between two groups, those with and without satisfaction by PGIC. The degrees of separation of the two groups were evaluated by ROC analyses. The area under the curve is shown next to the name of each clinical scale.

Post-treatment changes (Δ) in 8 major clinical scales were compared between the two groups with and without satisfaction by PGIC. The degree of separation of the two groups by each clinical scale was evaluated by ROC analyses (Fig 2). The AUC was highest by ΔPI-NRS (AUC = 0.770), followed by ΔHADS-dep (AUC = 0.733), ΔEQ5D (AUC = 0.686), and ΔLocomo 25 (AUC = 0.648).

**Fig 3:**
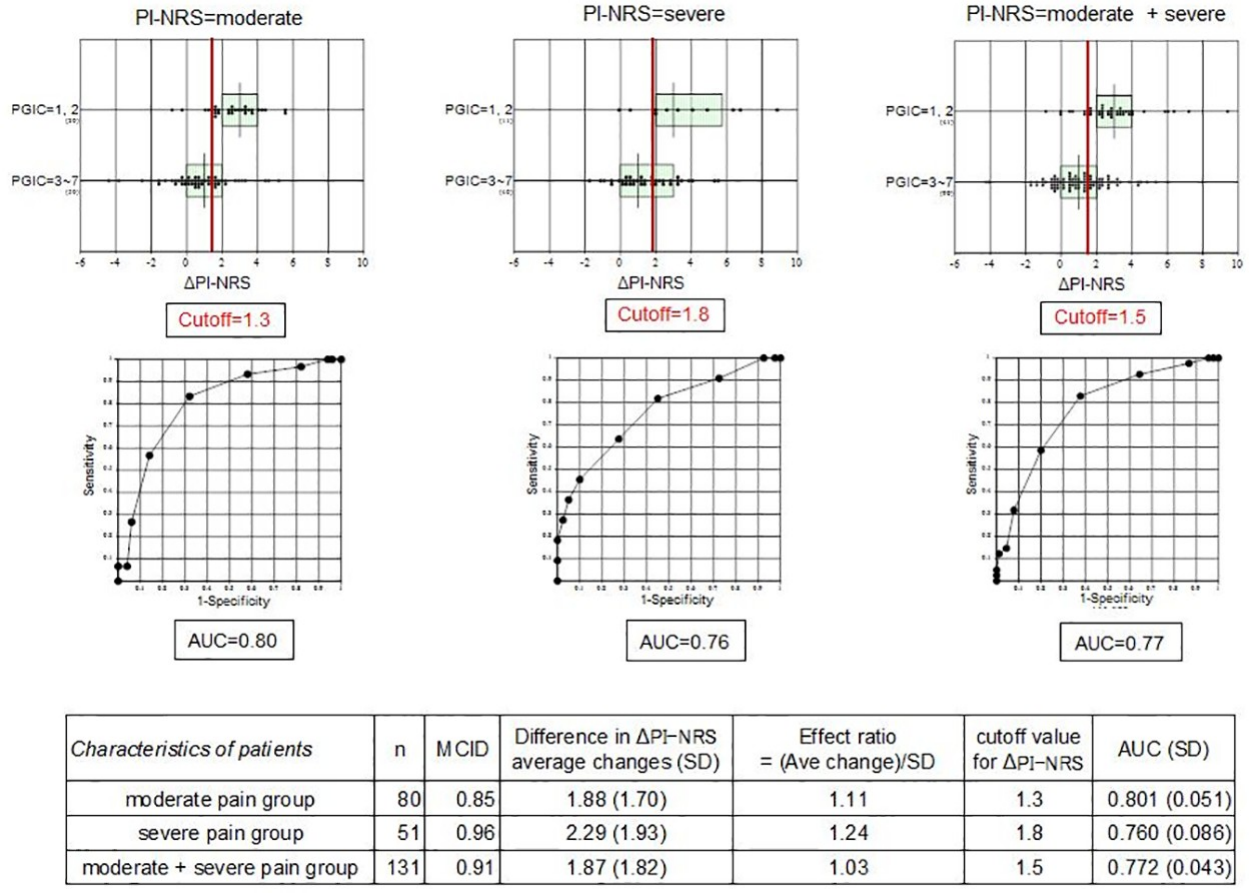
Minimal clinically important difference (MCID) and cutoff values for Δ Pain Intensity Numerical Rating Scale (PI-NRS) according to the baseline severity of PI-NRS. ΔPI-NRS was partitioned into two groups by the level of patient’s global impression of change (1–2 vs. 3–7). Optimal cutoff level was estimated as the ΔPI-NRS value at which sensitivity and specificity were equal. This analysis was done in three ways by subgrouping patients according to pre-treatment severity of PI-NRS: moderate, severe, and moderate + severe.

The optimal cutoff value was estimated as the ΔPI-NRS value at which sensitivity and specificity are equal. For the moderate, severe, and moderate + severe pain groups, the cutoff values for ΔPI-NRS were 1.3, 1.8, and 1.5, respectively (Fig 3). Because the PI-NRS takes an integer value between 0 to 10, these cutoff values can be rounded up to 2 for practical use regardless of the pre-treatment pain severity: i.e., when ΔPI-NRS ≥2, it is appropriate to consider that the patients felt much improved or improved (PGIC of 1 or 2). However, the MCID based on the inherent variability of ΔPI-NRS was 0.553 for the moderate pain group, 0.596 for the severe pain group, and 0.512 for the moderate + severe pain group (Fig 3). It is notable that the cutoff values shown above all exceeded the respective MCID values. As a whole, those MCID values for ΔPI-NRS can be rounded up to 1 for practical use regardless of the pretreatment level of pain.

### 2.2. Relationship of ΔPI-NRS with changes in other clinical scales

#### 2.2.1. Spearman’s correlation coefficients

To examine correlation among changes in the clinical scales associated with improvement in pain, i.e., ΔPI-NRS and ΔPDAS, ΔHADS (anxiety and depression), ΔPCS (rumination, magnification, and helplessness), ΔPSEQ, ΔAIS, ΔLocomo 25, and ΔEQ5D (Q1: pain/discomfort, Q2: anxiety/depression, Q3: mobility, Q4: self-care, Q5: usual activities), Spearman’s correlation coefficients (rS) were computed as shown in Table 6. By setting the effect size of rS = 0.30 as moderate and 0.50 as strong [6], |rS|≥0.3 is highlighted by bold font and |rS|≥0.5 by orange background color in this table.

**Table 6:** Spearman correlation coefficient between ΔPI-NRS and changes in other clinical scales.

	ΔPI−NRS	**ΔPDAS**	ΔHADSanx	ΔHADSdep	**ΔPCS**	ΔPCS_rum	ΔPCSmag	ΔPCSast	**ΔEQ5D**	ΔEQ5D1	ΔEQ5D2	ΔEQ5D3	ΔEQ5D4	ΔEQ5D5	**ΔPSEQ**	**ΔAIS**	**ΔLocomo 25**
ΔPI−NRS		**-0.43**	-0.27	**-0.31**	**-0.31**	-0.19	-0.23	**-0.34**	**0.34**	**0.44**	0.10	0.21	0.24	0.04	0.30	**-0.30**	**-0.37**
**ΔPDAS**	**-0.43**		0.28	**0.40**	**0.48**	**0.35**	0.28	**0.49**	**-0.51**	**-0.42**	**-0.31**	**-0.35**	**-0.35**	-0.21	**-0.42**	**0.41**	**0.67**
ΔHADSanx	-0.27	0.28		**0.44**	**0.34**	0.29	0.24	**0.31**	**-0.33**	-0.26	-0.24	-0.19	-0.21	-0.26	-0.24	0.28	0.27
ΔHADSdep	**-0.31**	**0.40**	**0.44**		**0.38**	0.27	0.23	**0.43**	**-0.38**	**-0.39**	-0.24	-0.24	-0.19	**-0.31**	**-0.37**	**0.39**	**0.38**
**ΔPCS**	**-0.31**	**0.48**	**0.34**	**0.38**		**0.78**	**0.78**	**0.88**	**-0.54**	**-0.37**	**-0.31**	**-0.39**	**-0.38**	**-0.32**	**-0.42**	**0.39**	**0.54**
ΔPCSrum	-0.19	**0.35**	0.29	0.27	**0.78**		**0.50**	**0.52**	**-0.40**	-0.20	**-0.35**	**-0.32**	-0.28	-0.26	**-0.42**	**0.35**	**0.42**
ΔPCSmag	-0.23	0.28	0.24	0.23	**0.78**	**0.50**		**0.56**	**-0.35**	-0.30	-0.13	-0.22	-0.26	-0.25	-0.19	0.20	**0.36**
ΔPCSast	**-0.34**	**0.49**	**0.31**	**0.43**	**0.88**	**0.52**	**0.56**		**-0.54**	**-0.39**	-0.29	**-0.41**	**-0.38**	-0.26	**-0.43**	**0.39**	**0.54**
**ΔEQ5D**	**0.34**	**-0.51**	**-0.33**	**-0.38**	**-0.54**	**-0.40**	**-0.35**	**-0.54**		**0.55**	**0.34**	**0.57**	**0.78**	**0.52**	**0.51**	**-0.40**	**-0.50**
ΔEQ5D1	**0.44**	**-0.42**	-0.26	**-0.39**	**-0.37**	-0.20	-0.30	**-0.39**	**0.55**		0.16	0.28	0.24	0.18	0.26	-0.29	**-0.40**
ΔEQ5D2	0.10	**-0.31**	-0.24	-0.24	**-0.31**	**-0.35**	-0.13	-0.29	**0.34**	0.16		0.09	0.16	0.23	**0.40**	-0.10	**-0.34**
ΔEQ5D3	0.21	**-0.35**	-0.19	-0.24	**-0.39**	**-0.32**	-0.22	**-0.41**	**0.57**	0.28	0.09		**0.31**	0.22	**0.31**	**-0.34**	**-0.44**
ΔEQ5D4	0.24	**-0.35**	-0.21	-0.19	**-0.38**	-0.28	-0.26	**-0.38**	**0.78**	0.24	0.16	**0.31**		0.28	**0.39**	**-0.33**	**-0.31**
ΔEQ5D5	0.04	-0.21	-0.26	**-0.31**	**-0.32**	-0.26	-0.25	-0.26	**0.52**	0.18	0.23	0.22	0.28		0.26	-0.26	-0.28
**ΔPSEQ**	**0.30**	**-0.42**	-0.24	**-0.37**	**-0.42**	**-0.42**	-0.19	**-0.43**	**0.51**	0.26	**0.40**	**0.31**	**0.39**	0.26		**-0.46**	**-0.49**
**ΔAIS**	**-0.30**	**0.41**	0.28	**0.39**	**0.39**	**0.35**	0.20	**0.39**	**-0.40**	-0.29	-0.10	**-0.34**	**-0.33**	-0.26	**-0.46**		**0.46**
**Δlocomo 25**	**-0.37**	**0.67**	0.27	**0.38**	**0.54**	**0.42**	**0.36**	**0.54**	**-0.50**	**-0.40**	**-0.34**	**-0.44**	**-0.31**	-0.28	**-0.49**	**0.46**	

PI-NRS, Pain Intensity Numerical Rating Scale; PDAS, Pain Disability Assessment Scale; HADS, Hospital Anxiety and Depression Scale; PCS, Pain Catastrophizing Scale; EQ-5D, EuroQoL 5 Dimension; PSEQ, Pain SelfEfficacy Questionnaire; Athens Insomnia Scale.

Moderate to strong correlations were frequently observed among ΔPI-NRS and other clinical scales (Table 6). Notably strong correlations of ΔPI-NRS were observed with ΔPDAS, ΔPSEC, ΔPCS, ΔAIS, ΔEQ5D, and ΔLocomo 25.

#### 2.2.2. Clinical utilities of post-treatment changes in the clinical scales

We applied the same method as was done for ΔPI-NRS to calculate MCIDs and cutoff values of ΔPDAS, ΔPSEC, ΔPCS, ΔAIS, ΔEQ5D, and ΔLocomo 25. The anchor for this analysis was the status of ΔPI-NRS partitioned at its cutoff value: ΔPI-NRS≥2 vs. ΔPI-NRS<2.

**Fig 4:**
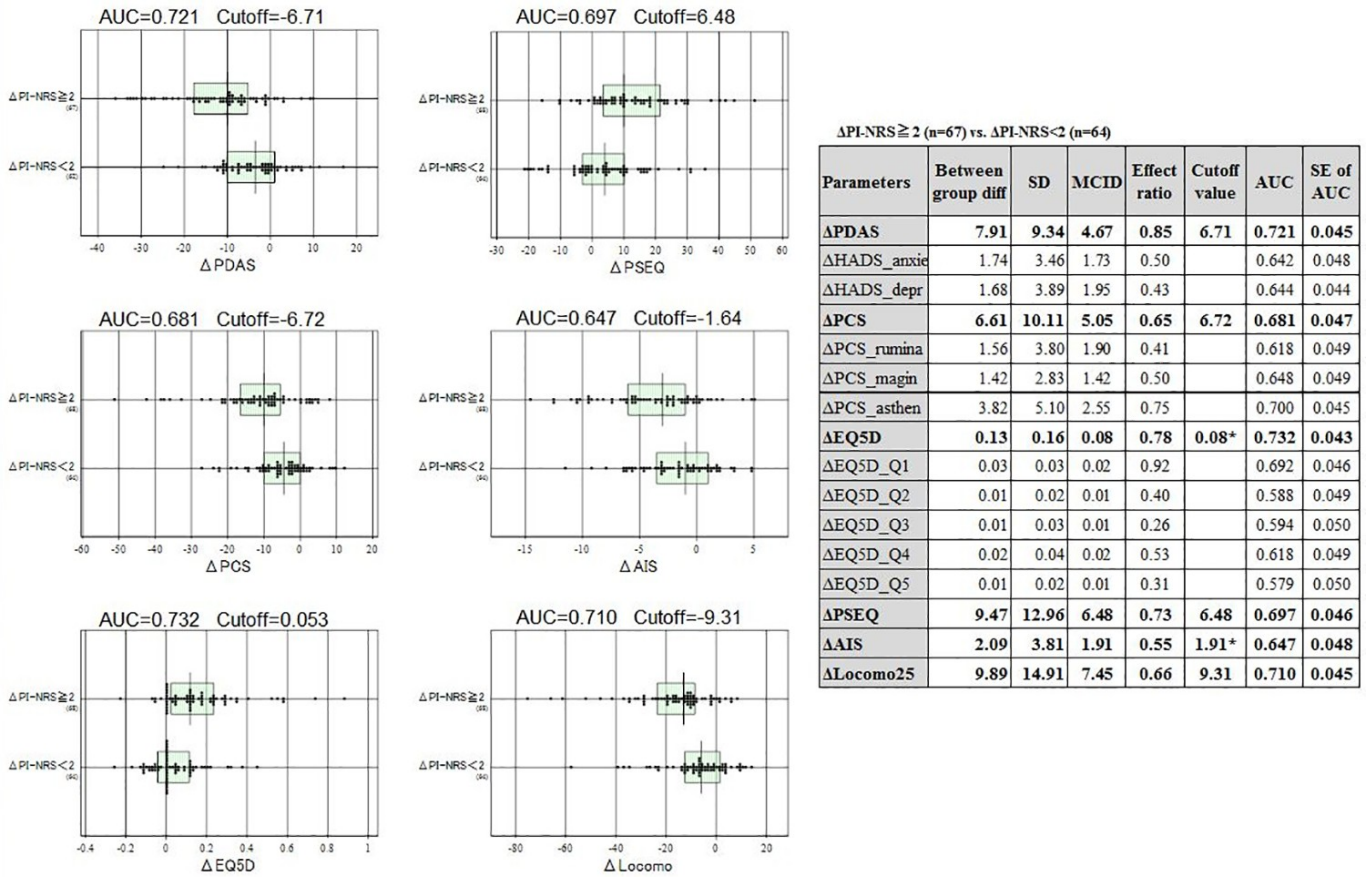
Associations of Δ Pain Intensity Numerical Rating Scale (PI-NRS) with post-treatment changes in 6 clinical scales. Distributions of post-treatment changes in the 6 clinical scales were compared between two groups partitioned at the cutoff value of ΔPI-NRS = 2.0. The degree of separation of the two groups is expressed as an area under curve (AUC) by the receiver operating characteristic analysis and shown on top of each graph, together with the optimal cutoff value for the distinction. The table on the right shows a list of the AUCs and standard error of the AUCs that were determined for all clinical parameters by use of the same analysis described above.

The MCID and cutoff value in predicting ΔPI-NRS≥2 status were 4.67, 6.71 for ΔPDAS (score range: 0−60); 6.48, 6.48 for ΔPSEC (0−60); 5.05, 6.71 (0−52) for ΔPCS; 1.9, 1.64 for ΔAIS (0−24); 0.08, 0.053 for ΔEQ5D (0−1.0); and 7.5, 9.31 for ΔLocomo 25 (0−100) (Fig 4). These cutoff values exceeded the respective MCID except for ΔAIS and ΔEQ5D, indicating that a change in their score above the cutoff value can be interpreted as clinically relevant. The imbalance of the MCID and cutoff value observed for ΔAIS and ΔEQ5D were caused by unequal scatter of values between the two groups resulting in unreliable cutoff values. Therefore, their cutoff values were raised to the level of respective MCIDs to avoid false-positive interpretation of their changes.

## Discussion

It is most important to set the objective goal in clinical after CLBP treatment because it is difficult that we make the patients with completely no pain. The patients with CLBP commonly have not only lumber dysfunction but also physical disability and psychosocial issues [1]. The quantification of the outcome of CLBP treatment using some simple clinical scale is crucical in the clinical management [12, 15]. To reveal the cutoff points and the MCID for ΔPI-NRS based on PGIC in CLBP are the most simple and acceptable for the medical staff when we quantify the treatment goal [17–25].

We comprehensibly evaluated the physical disability and psychosocial conditions in patients with CLBP using self-administered multifaceted measures because these patients have multidimensional musculoskeletal, social, mental and cognitive disorders, and other issues [11–12, 21, 29, 30]. However, we cannot tell how much of an improvement in each score can be regarded as clinically meaningful [12]. The lack of objective cutoff values of improvement in these clinical scales restricts proper interpretation of the scores when conducting research into treatment outcomes in CLBP [12, 31].

At our pain management centers, we evaluate the intensity of pain in patients with CLBP using the PI-NRS, PDAS, HADS, PCS, PSEQ, EQ-5D, AIS, and Locomo 25, which cover multifaceted issues [9, 15, 21, 32–33]. If these complex clinical scales can be interpreted in a unified way, evaluation of a patient becomes less difficult and more objective, and current efforts of the medical staff will be eased. In addition, the availability of objective cutoff values for these clinical scales for use as treatment goals can widely expand the number of clinical facilities capable of managing pain in CLBP patients.

When both clinicians and investigators calculate cutoff values for these measures, we think the patient’s perspective on the meaning of changes following treatment will become core outcome measures [28, 34–36]. A commonly used method to determine thresholds for patient-perceived meaningful change is to compare changes in pain scores with patients’ global ratings of the magnitude of change [28, 37–38]. We thus focused on ΔPI-NRS as the most important assessment anchoring treatment because the threshold of change in PI-NRS, i.e., ΔPI-NRS, following treatment is generally considered to be quite useful for judging the effect of therapy, and the threshold is the universal indicator of change in individual patients [3–4, 16, 39]. In the present study, we sought to determine the cutoff points and the MCID for ΔPI-NRS based on PGIC in CLBP [18]. We revealed that the MCID of ΔPI-NRS was 1 regardless of the level of pre-treatment pain in CLBP patients, and this led to the determination of a treatment goal of 2 for ΔPI-NRS as the cutoff value in CLBP.

Several papers have reported cutoff values for chronic musculoskeletal pain [17, 39–40]. Salaffi et al. reported the MCID in chronic musculoskeletal pain intensity measured on a NRS [40]. A change in the NRS score of −2.0 and a percent change score of −33.0% were best associated with the concept of “much better” improvement. Although the database used in their report did not contain CLBP patients, they reported the important threshold score of 2 for NRS change, which was the same as our cutoff value of 2.

In the measurement of pain relief in patients with trigeminal neuralgia, Sandhu et al. reported that the MCIDs for the 3 domains of the Brief Pain Inventory-Facial were 57% and 28% improvement in pain intensity for the worst and average pain, respectively, 75% improvement in interference with general activities of daily living, and 62% improvement in interference with facial activities of daily living [39]. Another paper defined the MCID for grade I degenerative lumbar spondylolisthesis following lumbar surgery [41]. The MCID values were 1.6 points for NRS-back pain, 1.7 points for NRS-leg pain, 14.3 points for the Oswestry Disability Index, and 0.2 points for the EQ-5D. These previous papers reported similar scores for cutoff values and MCIDs even if the target disease was different from that in the present study. We thus think a ΔPI-NRS of 2 is the key threshold score in evaluating treatment outcome of CLBP patients.

As a next step, the cutoff values and the MCID were also determined for ΔPDAS, ΔPSEC, ΔPCS, ΔAIS, ΔEQ5D, and ΔLocomo 25 based on ΔPI-NRS (ΔPI-NRS≥2 or ΔPI-NRS<2) in the present study. To our best knowledge, this is the first report to reveal a meaningful threshold value for the treatment goal and the cutoff value for treatment effect for each of these clinical scales. Only limited cutoff values were reported previously, and no reports showed the meaningful threshold change for treatment. Yamashiro et al. reported that the cutoff value for the PDAS was 10. A score of over 41 points in the PSEQ means the patient has high self-efficacy, whereas that below 20 points indicates low self-efficacy [15]. Sullivan et al. reported that the cutoff value in the PCS was 30 [14]. Four to five points in the AIS suggests a sleep disorder, and over 6 points suggest the high possibility of a sleep disorder. Over 16 in the Locomo 25 indicates locomotive syndrome [10]. We established objective and meaningful threshold scores for ΔPDAS of 6.71, ΔPSEC of 6.48, ΔPCS of 6.71, ΔAIS of 1.9, ΔEQ5D of 0.08, and ΔLocomo 25 of 9.31 in the treatment of CLBP patients.

From another point of view, when medical staff observe a ΔPI-NRS of ≥2 in patients after treatment, we can interpret this to indicate that the CLBP patients have gotten well with satisfaction and improvement in the multifaceted scores measuring life disabilities, patient confidence with ongoing pain, pain catastrophizing, sleep disorder, QOL, and musculoskeletal disorders.

In summary, as clinically significant threshold and treatment target in CLBP treatment, we revealed a ΔPI-NRS of ≥2 and the threshold scores for ΔPDAS of 6.71, ΔPSEC of 6.48, ΔPCS of 6.71, ΔAIS of 1.9, ΔEQ5D of 0.08, and ΔLocomo 25 of 9.31. We propose those definitive target scores as directly correlating with PGIC for use all of the medical staff in the management of CLBP patients.

A limitation of this study is that the score cannot be generalized to all CLBP patients because the data were taken only from a short 3-month follow-up period and from CLBP patients treated in only two pain management centers. Second, it is inevitable to have some degree of inconsistencies between MCID score and cutoff values in some clinical scales like AIS and EQ5D, although we derived MCID in the same way as in the previous reports possibly due to insufficient number of cases enrolled. In the present study we adopted either the MCID or the cutoff value with higher score as indicative of more clinical utility.

In conclusion, we revealed a new indicator in the evaluation of CLBP treatment. A ΔPI-NRS value of 2 is the key score in CLBP treatment.

## Supporting information

S1 Fig (DOCX)

## References

[1] Chou R, Shekelle P. Will this patient develop persistent disabling low back pain? JAMA 2010;303:1295–302. 10.1001/jama.2010.344 20371789

[2] WHO. The burden of musculoskeletal conditions at the start of the new millennium. World Health Organ Tech Rep Ser. 2003;919:1–218. 14679827

[3] Chauny JM, Paquet J, Lavigne G, Marquis M, Daoust R. Evaluating acute pain intensity relief: challenges when using an 11-point numerical rating scale. PAIN 2016;157:355–60. 10.1097/j.pain.0000000000000382 26447700

[4] Farrar JT, Young JP Jr, LaMoreaux L, Werth JL, Poole RM. Clinical importance of changes in chronic pain intensity measured on an 11-point numerical pain rating scale. PAIN 2001;94:149–58. 10.1016/s0304-3959(01)00349-9 11690728

[5] Parker SL, Mendenhall SK, Shau DN, Adogwa O, Anderson WN, Devin CJ, et al Minimum clinically important difference in pain, disability, and quality of life after neural decompression and fusion for same-level recurrent lumbar stenosis: understanding clinical versus statistical significance. J Neurosurg Spine 2012;16:471–8. 10.3171/2012.1.SPINE11842 22324801

[6] Balestroni G, Bertolotti G. EuroQol-5D (EQ-5D): an instrument for measuring quality of life. Monaldi Arch Chest Dis 2012;78:155–9. 10.4081/monaldi.2012.121 23614330

[7] Dolan P. Modeling valuations for EuroQol health states. Med Care 1997;35:1095–108. 10.1097/00005650-199711000-00002 9366889

[8] Kim HJ, Kwon OH, Chang BS, Lee CK, Chun HJ, Yeom JS. Change in pain catastrophizing in patients with lumbar spinal surgery. Spine J 2018;18:115–121. 10.1016/j.spinee.2017.06.028 28669860

[9] Chiarotto A, Boers M, Deyo RA, Buchbinder R, Corbin TP, Costa LOP, et al Core outcome measurement instruments for clinical trials in nonspecific low back pain. PAIN 2018;159:481–95. 10.1097/j.pain.0000000000001117 29194127 PMC5828378

[10] Nakamura K, Ogata T. Locomotive syndrome: definition and management. Clin Rev Bone Miner Metab 2016;14:56–67. 10.1007/s12018-016-9208-2 27375370 PMC4906066

[11] Nicholas MK. The pain self-efficacy questionnaire: Taking pain into account. Eur J Pain 2007;11:153–63. 10.1016/j.ejpain.2005.12.008 16446108

[12] Nicholas MK, Asghari A, Blyth FM. What do the numbers mean? Normative data in chronic pain measures. PAIN 2008;134:158–73. 10.1016/j.pain.2007.04.007 17532138

[13] Soldatos CR, Dikeos DG, Paparrigopoulos TJ. Athens Insomnia Scale: validation of an instrument based on ICD-10 criteria. J Psychosom Res 2000;48:555–60. 10.1016/s0022-3999(00)00095-7 11033374

[14] Sullivan MJ, Thorn B, Haythornthwaite JA, Keefe F, Martin M, Bradley LA, et al Theoretical perspectives on the relation between catastrophizing and pain. Clin J Pain 2001;17:52–64. 10.1097/00002508-200103000-00008 11289089

[15] Yamashiro K, Arimura T, Iwaki R, Jensen MP, Kubo C, Hosoi M. A multidimensional measure of pain interference: Reliability and validity of the pain disability assessment scale. Clin J Pain 2011;27:338–43. 10.1097/AJP.0b013e318204858a 21178590

[16] Strong J, Unruh AM, Wright A, Baxter GD. Pain: A textbook for therapists. London: Churchill Livingstone; 2001 pp. 480.

[17] Boonstra AM, Schiphorst Preuper HR, Balk GA, Stewart RE. Cut-off points for mild, moderate, and severe pain on the visual analogue scale for pain in patients with chronic musculoskeletal pain. PAIN 2014;155:2545–50. 10.1016/j.pain.2014.09.014 25239073

[18] Emshoff R, Bertram S, Emshoff I. Clinically important difference thresholds of the visual analog scale: A conceptual model for identifying meaningful intraindividual changes for pain intensity. PAIN 2011;152:2277–82. 10.1016/j.pain.2011.06.003 21726939

[19] ten Klooster PM, Drossaers-Bakker KW, Taal E, van de Laar MA. Patient-perceived satisfactory improvement (PPSI): interpreting meaningful change in pain from the patient's perspective. PAIN 2006;121:151–7. 10.1016/j.pain.2005.12.021 16472915

[20] van der Roer N, Ostelo RW, Bekkering GE, van Tulder MW, de Vet HC. Minimal clinically important change for pain intensity, functional status and general health status in patients with nonspecific low back pain. Spine (Phila Pa 1976) 2006;31:578–82. 16508555 10.1097/01.brs.0000201293.57439.47

[21] Hayashi K, Arai YC, Ikemoto T, Nishihara M, Suzuki S, Hirakawa T, et al Predictive factors for the outcome of multidisciplinary treatments in chronic low back pain at the first multidisciplinary pain center of Japan. J Phys Ther Sci 2015;27:2901–5. 10.1589/jpts.27.2901 26504321 PMC4616122

[22] Keller S, Bann CM, Dodd SL, Schein J, Mendoza TR, Cleeland CS. Validity of the brief pain inventory for use in documenting the outcomes of patients with noncancer pain. Clin J Pain 2004;20:309–18. 10.1097/00002508-200409000-00005 15322437

[23] Jaeschke R, Singer J, Guyatt GH. Measurement of health status. Ascertaining the minimal clinically important difference. Control Clin Trials 1989;10:407–15. 10.1016/0197-2456(89)90005-6 2691207

[24] MacDowall A, Skeppholm M, Robinson Y, Olerud C. Validation of the visual analog scale in the cervical spine. J Neurosurg Spine 2018;28:227–35. 10.3171/2017.5.SPINE1732 29243996

[25] Parker SL, Mendenhall SK, Shau D, Adogwa O, Cheng JS, Anderson WN, et al Determination of minimum clinically important difference in pain, disability, and quality of life after extension of fusion for adjacent-segment disease. J Neurosurg Spine 2012;16:61–7. 10.3171/2011.8.SPINE1194 21962034

[26] Cohen J. A power primer. Psychol Bull 1992;112:155–9. 10.1037//0033-2909.112.1.155 19565683

[27] Copay AG, Subach BR, Glassman SD, Polly DW Jr, Schuler TC. Understanding the minimum clinically important difference: a review of concepts and methods. Spine J 2007;7:541–6. 10.1016/j.spinee.2007.01.008 17448732

[28] Crosby RD, Kolotkin RL, Williams GR. Defining clinically meaningful change in health-related quality of life. J Clin Epidemiol 2003;56:395–407. 10.1016/s0895-4356(03)00044-1 12812812

[29] Lee JS, Hobden E, Stiell IG, Wells GA. Clinically important change in the visual analog scale after adequate pain control. Acad Emerg Med 2003;10:1128–30. 10.1111/j.1553-2712.2003.tb00586.x 14525749

[30] Nicholas MK, Asghari A, Blyth FM, Wood BM, Murray R, McCabe R, et al Long-term outcomes from training in self-management of chronic pain in an elderly population: a randomized controlled trial. PAIN 2017;158:86–95. 10.1097/j.pain.0000000000000729 27682207

[31] Liu S, Schwab F, Smith JS, Klineberg E, Ames CP, Mundis G, et al Likelihood of reaching minimal clinically important difference in adult spinal deformity: a comparison of operative and nonoperative treatment. Ochsner J 2014;14:67–77. 24688336 PMC3963055

[32] Iizuka Y, Iizuka H, Mieda T, Tsunoda D, Sasaki T, Tajika T, et al Prevalence of chronic nonspecific low back pain and its associated factors among middle-aged and elderly people: an analysis based on data from a musculoskeletal examination in Japan. Asian Spine J 2017;11:989–97. 10.4184/asj.2017.11.6.989 29279756 PMC5738322

[33] Stratford PW, Binkley JM, Riddle DL, Guyatt GH. Sensitivity to change of the Roland-Morris Back Pain Questionnaire: part 1. Phys Ther 1998;78:1186–96. 10.1093/ptj/78.11.1186 9806623

[34] Beaton DE, Boers M, Wells GA. Many faces of the minimal clinically important difference (MCID): a literature review and directions for future research. Curr Opin Rheumatol 2002;14:109–14. 10.1097/00002281-200203000-00006 11845014

[35] Bombardier C, Hayden J, Beaton DE. Minimal clinically important difference: low back pain. Outcome measures. J Rheumatol 2001;28:431–8. 11246692

[36] Copay AG, Glassman SD, Subach BR, Berven S, Schuler TC, Carreon LY. Minimum clinically important difference in lumbar spine surgery patients: a choice of methods using the Oswestry Disability Index, Medical Outcomes Study questionnaire Short Form 36, and pain scales. Spine J 2008;8:968–74. 10.1016/j.spinee.2007.11.006 18201937

[37] Tetreault L, Nouri A, Kopjar B, Côté P, Fehlings MG. The minimum clinically important difference of the Modified Japanese Orthopaedic Association Scale in patients with degenerative cervical myelopathy. Spine (Phila Pa 1976) 2015;40:1653–9. 26502097 10.1097/BRS.0000000000001127

[38] Wells G, Beaton D, Shea B, Boers M, Simon L, Strand V, et al Minimal clinically important differences: review of methods. J Rheumatol 2001;28:406–12. 11246688

[39] Sandhu SK, Halpern CH, Vakhshori V, Mirsaeedi-Farahani K, Farrar JT, Lee JY. Brief pain inventory—facial minimum clinically important difference. J Neurosurg 2015;122:180–90. 10.3171/2014.8.JNS132547 25361481

[40] Salaffi F, Stancati A, Silvestri CA, Ciapetti A, Grassi W. Minimal clinically important changes in chronic musculoskeletal pain intensity measured on a numerical rating scale. Eur J Pain 2004;8:283–91. 10.1016/j.ejpain.2003.09.004 15207508

[41] Asher AL, Kerezoudis P, Mummaneni PV, Bisson EF, Glassman SD, Foley KT, et al Defining the minimum clinically important difference for grade I degenerative lumbar spondylolisthesis: insights from the Quality Outcomes Database. Neurosurg Focus 2018;44:E2. 10.3171/2017.10.FOCUS17554 29290132

