# Supplementary figures and images for: Clinically significant changes in pain along the Pain Intensity Numerical Rating Scale in patients with chronic low back pain
Source: PLoS One. 2020 Mar 3;15(3):e0229228. doi: 10.1371/journal.pone.0229228 (PMC7053735; doi:10.1371/journal.pone.0229228)

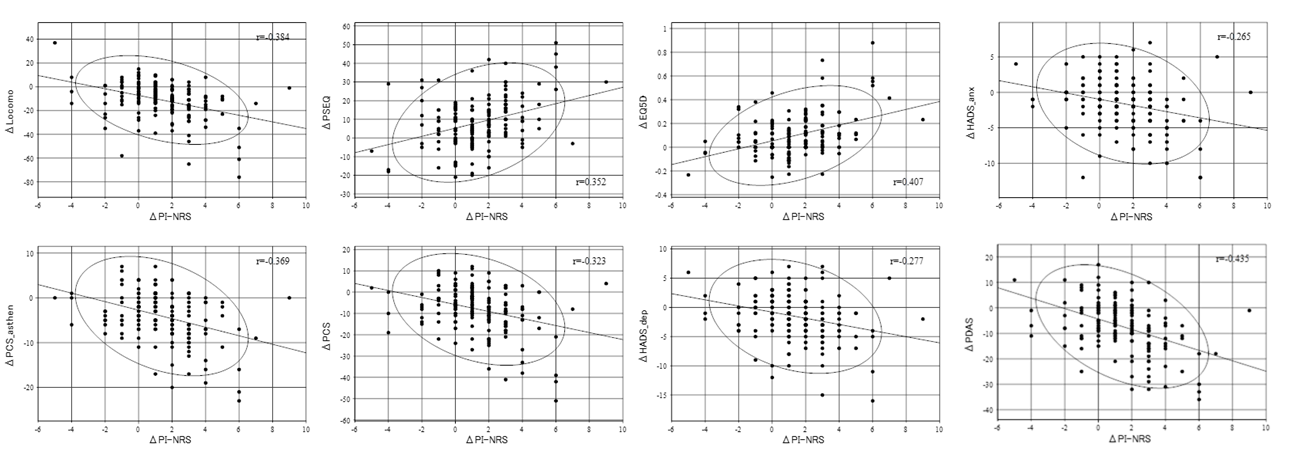

Supplement: S1 Fig — (DOCX) [file pone.0229228.s001.docx]
